# Comparative Response of the Hepatic Transcriptomes of Domesticated and Wild Turkey to Aflatoxin B_1_

**DOI:** 10.3390/toxins10010042

**Published:** 2018-01-13

**Authors:** Kent M. Reed, Kristelle M. Mendoza, Juan E. Abrahante, Roger A. Coulombe

**Affiliations:** 1 Department of Veterinary and Biomedical Sciences, College of Veterinary Medicine, University of Minnesota, Saint Paul, MN 55108, USA; mendo008@umn.edu; 2 University of Minnesota Informatics Institute, University of Minnesota, Minneapolis, MN 55455, USA; abrah023@umn.edu; 3 Department of Animal, Dairy and Veterinary Sciences, College of Agriculture, Utah State University, Logan, UT 84322, USA; roger@usu.edu

**Keywords:** aflatoxin B_1_, domesticated turkey, wild turkey, liver, RNA-seq, differential expression, Results of this study support the hypothesis that the greater ability of wild turkeys to detoxify AFB_1_ is related to higher constitutive expression of *GSTA3* coupled with an inherited (genetic) difference in functional expression in domesticated birds. Key differences in *GSTA3* expression between the Eastern wild and domesticated turkeys is not unique to these genetic lines but is a broader phenomenon indicating lower fitness in domesticated birds. Results of RNA-seq analysis emphasize the differential response of these genetically distinct birds, demonstrating significant differences in expression of Phase I and Phase II genes and in genes important in toxic response.

## Abstract

The food-borne mycotoxin aflatoxin B_1_ (AFB_1_) poses a significant risk to poultry, which are highly susceptible to its hepatotoxic effects. Domesticated turkeys (*Meleagris gallopavo*) are especially sensitive, whereas wild turkeys (*M. g. silvestris*) are more resistant. AFB_1_ toxicity entails bioactivation by hepatic cytochrome P450s to the electrophilic exo-AFB_1_-8,9-epoxide (AFBO). Domesticated turkeys lack functional hepatic GST-mediated detoxification of AFBO, and this is largely responsible for the differences in resistance between turkey types. This study was designed to characterize transcriptional changes induced in turkey livers by AFB_1_, and to contrast the response of domesticated (susceptible) and wild (more resistant) birds. Gene expression responses to AFB_1_ were examined using RNA-sequencing. Statistically significant differences in gene expression were observed among treatment groups and between turkey types. Expression analysis identified 4621 genes with significant differential expression (DE) in AFB_1_-treated birds compared to controls. Characterization of DE transcripts revealed genes dis-regulated in response to toxic insult with significant association of Phase I and Phase II genes and others important in cellular regulation, modulation of apoptosis, and inflammatory responses. Constitutive expression of *GSTA3* was significantly higher in wild birds and was significantly higher in AFB_1_-treated birds when compared to controls for both genetic groups. This pattern was also observed by qRT-PCR in other wild and domesticated turkey strains. Results of this study emphasize the differential response of these genetically distinct birds, and identify genes and pathways that are differentially altered in aflatoxicosis.

## 1. Introduction

Aflatoxin B_1_ (AFB_1_) is a ubiquitous hepatotoxic, hepatocarcinogenic, and immunosuppressive mycotoxin. Poultry and other livestock are exposed to AFB_1_ by consuming contaminated feed. Many agricultural feed commodities (corn, cottonseed, peanuts, and sorghum) and other foods (figs, tree nuts, and spices) are at especially high risk of being contaminated [1]. AFB_1_ is practically unavoidable in most feed ingredients, especially corn [2,3,4], and is expected to concomitantly increase with global climate change [5]. Approximately 25% of the world’s annual food supply is contaminated with mycotoxins, and losses attributed to AFB_1_ are significant to the poultry industry [1].

Poultry are among the most sensitive animals to the toxic effects of AFB_1_ [6,7]. Domesticated turkeys are among the most sensitive species [8], but wild turkeys are more resistant [9]. Turkey sensitivity is historically important because it was instrumental in the discovery of AFB_1_ as being responsible for the deaths of domestic turkeys in Europe due to “Turkey X Disease” that was traced to contaminated feed [10]. AFB_1_ is a potent immunotoxin acting to suppress cell-mediated, humoral, and phagocytic functions in chickens and turkeys [11,12,13]. As a result, it has a wide array of toxic effects, including; reduced feed intake, weight gain, and feed efficiency, and increased mortality, hepatotoxicity, GI hemorrhaging, and susceptibility to bacterial and viral diseases. Embryonic exposure to AFB_1_ produces dose-related DNA damage [14] and compromised immune response through suppression of humoral and cellular immunity making hatched chicks more susceptible to disease [13]. Thus, in addition to being a potent natural toxin, AFB_1_ is a powerful “force-multiplier”, amplifying adverse effects of other agents that are detrimental to poultry health.

Aflatoxin B_1_ toxicity requires bioactivation by hepatic cytochrome P450s (CYPs) to the electrophilic exo-AFB_1_-8,9-epoxide (AFBO). In the absence of GST activity, AFBO can form adducts that bind to DNA, RNA and other macromolecules, causing immunotoxicity, mutations, and aflatoxicosis [15]. The extreme sensitivity of domesticated turkeys to AFB_1_ is associated with efficient epoxidation by cytochromes P4501A5 and 3A37 [16], both of which have been cloned, heterologously expressed and functionally characterized [8,17]. Using anti-peptide antibodies, P4501A5 was found to be the dominant bioactivating and metabolizing enzyme at environmentally relevant AFB_1_ concentrations in turkey liver [8].

While P450-mediated bioactivation plays an important role, the principal determinant of response to AFB_1_ is the efficiency of detoxification by hepatic glutathione S-transferases (GSTs), most notably alpha class (GSTAs) [18]. The α-GST cluster in turkeys includes six genes, *GSTA1.1*-*A1.3*, *GSTA2*, *GSTA3*, and *GSTA4* [19,20]. Whereas, wild and heritage breed turkeys possess GST-mediated AFBO detoxification activity, livers from domestic turkeys lack detectable activity [20]. Thus, the most likely mechanism for the extreme sensitivity of domestic turkeys is dysfunction in hepatic GSTs, rendering them unable to detoxify AFB_1_ [21,22,23]. As a result, AFBO forms adducts, which can induce DNA mutations, block transcription and alter translation [24,25].

To understand the response of the domesticated turkey to AFB_1_ exposure, we initiated study of the hepatic transcriptome following dietary AFB_1_ challenge. Results of this study identified genes and gene pathways in the liver directly affected by AFB_1_ [26]. Functional analysis found transcripts significantly dis-regulated by toxicity and affecting pathways of cancer, apoptosis, cell cycle, and lipid regulation. These changes reflect the molecular mechanisms of inflammation, proliferation and liver damage in aflatoxicosis. This study was followed by analysis of spleen tissues from the same birds [27] that found short exposure to AFB_1_ suppressed innate immune transcripts, especially from antimicrobial genes that are indicative of either increased cytotoxic potential or activation-induced cell death in the spleen during aflatoxicosis.

To better examine the differences between wild and domesticated birds, we developed an *in ovo* exposure model to provide controlled AFB_1_ exposure to developing embryos [28]. RNA-seq analysis found AFB_1_ effects were dependent on both length of exposure and turkey type (domesticated vs. wild), confirming significant differences in the response to AFB_1_ attributed to genetic background [28]. Transcriptome responses to AFB_1_ occurred more rapidly in domesticated birds (1 day post-exposure), and led to the up regulation in cell cycle regulators, Nrf2-mediated response genes and coagulation factors. Expression changes in the embryonic liver also suggested cellular responses to oxidative stress and xenobiotics were initiated by AFB_1_ exposure. In contrast, the response in wild turkey embryos occurred later (five days post-exposure). Combined, these studies demonstrated that GST-mediated hepatic detoxification of AFBO is largely responsible for the differences in resistance between turkey types, but other processes and pathways (i.e., apoptosis, cellular regulation, immune responses) are also important. Whereas, understanding the effects of AFB_1_ on developing embryos is important in poultry production, the manifestation of AFB_1_ toxicity is likely to be different in more mature birds with fully developed gastrointestinal systems. The purpose of this study was to compare the hepatic transcriptome response to dietary AFB_1_ in juvenile (three weeks of age), susceptible (domesticated), and more resistant (wild) turkeys. We hypothesized that transcriptome responses in juvenile birds would reflect the more mature status of the gastrointestinal and antioxidant systems than those of embryos.

## 2. Results

Liver measurements were collected at the end of the exposure trial to characterize phenotypic effects of AFB_1_ toxicity. Livers of domesticated (DT) birds (average = 20.54 g) were nearly three times the mass of those from Eastern wild (EW) birds (8.3 g) primarily due to differences in body size (average = 1147.5 g and 396.1 g, respectively). Liver weights of AFB_1_ turkeys were smaller than those of the control (CNTL) groups. In DT, average liver mass at the conclusion of the trial ranged from 14.76 g to 23.98 g in the AFB_1_ group (mean = 20.02 ± 2.44 g) and from 17.39 g to 25.11 g in controls (21.00 ± 2.12 g). Although this difference in liver mass was not significant (*t*-test *p* = 0.1962), when corrected for body weight (% BW) the livers of birds from the AFB_1_-treated group were significantly smaller (*p* = 0.0098). Livers of AFB_1_-treated EW birds (7.19 ± 1.06 g) were similarly smaller than those of control birds (9.43 ± 1.11 g). This difference was significant for absolute mass (*p* = 0.0005) and nearly so for % BW (*p* = 0.0531).

Sequencing of RNA libraries produced over 195 M reads. The number of reads per library ranged from 10.8 M to 14.6 M (average 12.2 M, Table 1). After trimming and filtering, median Q scores were consistently high and ranged from 36.4 to 37.4 among the forward and reverse reads. The number of reads per treatment group was balanced and ranged from 11.5 to 12.7 M, with an average of 12.21 ± 0.5 M. Approximately 91% of the quality-trimmed reads mapped to the annotated turkey gene set (NCBI Annotation 101, Table 1). This percentage was consistent across treatment groups and the percentage of aligned read pairs exceeded 89.7% (average = 90.8%); the majority of reads (average = 85.3%) mapped concordantly (Table 1). Based on mapping, the estimated mean library insert was 191.7 bp.

**Figure 1:**
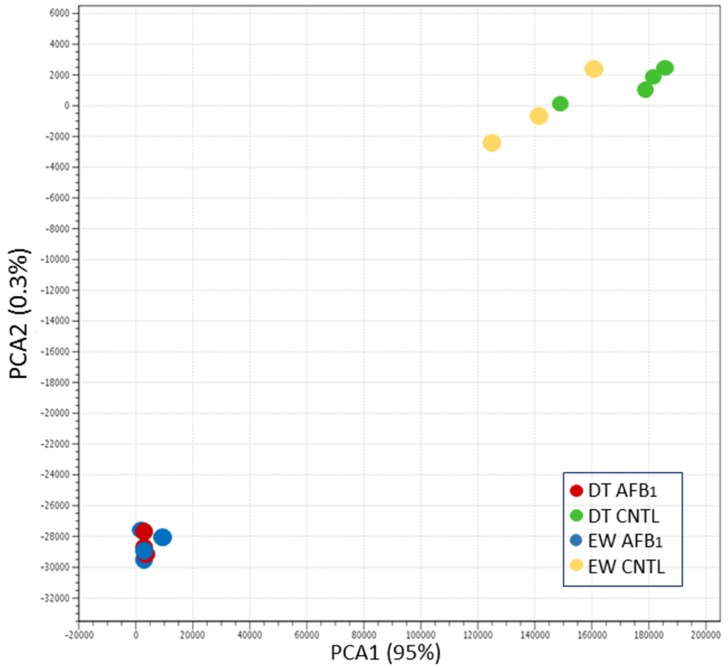
Principal component analysis (PCA) of normalized RNA-seq read counts. For each treatment group, sample to sample distances (within- and between-treatments) are illustrated on the first two principal components comprising approximately 95% of the variation.

Principal component analysis (PCA) of normalized read counts visualized variation among the treatment groups (Figure 1). Groups clustered distinctly according to treatment (AFB_1_ versus control) within the first two principal components, accounting for approximately 95% of the observed variation. Hierarchical clustering of groups by Euclidean distance reiterated the relationships shown by PCA (Figure S1). After segregating by AFB_1_ treatment, groups secondarily cluster by type (domesticated versus wild), with the exception of samples N3L (domesticated) and EW1L (wild) that clustered with the opposite bird type. Significant differences in overall gene counts among groups are shown in the heat map of co-expressed genes.

### 2.1. Gene Expression

Evidence of expression (mean mapped reads ≥ 1.0 in at least one treatment group) was detected for 19,764 genes (tRNAs excluded), with an average of 17,137.5 genes being detected per group (81.56% of the turkey gene set) (Table S1). Mean read depth was 394.8 reads per gene. When limited to an average number of mapped reads ≥ 3.0, the number of expressed genes ranged from 14,399 to 17,230 among treatment groups (average 15,979.25, Table 1). Distribution of unique and shared expressed genes is illustrated in Figure S2. A total of 14,373 genes (81.2%) was co-expressed among all groups, and the number of co-expressed genes within the EW and DT lines was 14,908 and 14,669, respectively. Each treatment group had distinct sets of uniquely expressed genes, but that number was considerably greater in the AFB_1_-treatment groups (2169, 12.3%) when compared to controls (300, 1.6%, Table 1 and Table S1).

### 2.2. Differential Transcriptomic Expression: AFB_1_ Effects

**Figure 2:**
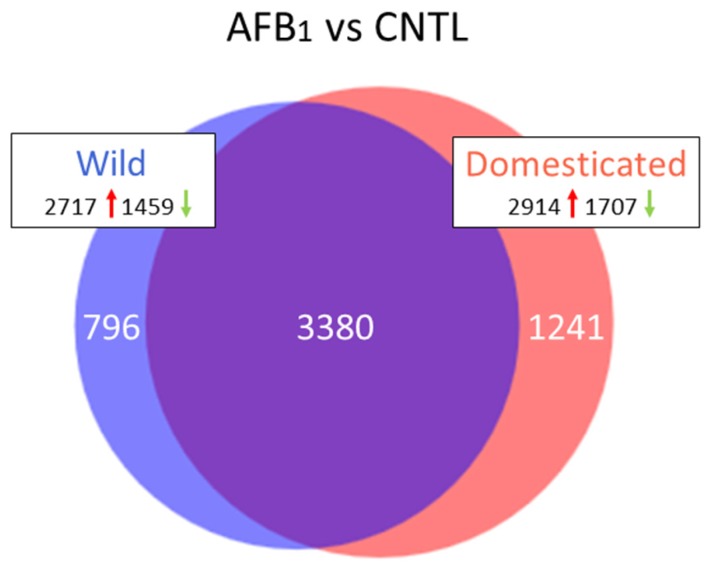
Distribution of differentially expressed genes in turkey. For each comparison, the number of genes with FDR *p*-value < 0.05 and |log_2_FC| > 2.0 shared or unique to each treatment are indicated in the Venn diagram. Circle size is proportional to the number of genes and direction of expression change (↑ or ↓) is given for the genes in each group.

Table S2 provides the full list of genes showing significant differential expression (DE). DE was observed for 9620 genes (FDR *p*-value < 0.05, log_2_FC = −9.670 to 9.358) in wild turkeys exposed to AFB_1_ when compared to control birds with 4176 genes having |log_2_FC| > 2.0 (Table 2). Similarly, 11,325 DE genes were observed for the AFB_1_-treated DT turkeys (FDR *p*-value < 0.05, log_2_FC = −14.133 to 12.676) with 4621 genes having |log_2_FC| > 2.0. The majority of DE genes (3380) were shared in both bird types, with 796 being unique to wild and 1241 unique to the domesticated birds (Figure 2). The majority of DE genes was up regulated by AFB_1_ treatment in both the wild (2717, 65%) and domesticated birds (2914, 70%). Of the 50 genes showing the greatest fold change with treatment, seven (*ANGPTL3* [angiopoietin-like 3], *GC* [group-specific component (vitamin D binding protein)], *LOC104911607* [uncharacterized ncRNA], *LOC100541166* [alpha-1-acid glycoprotein 2-like], *LOC100542070* [*SERPINA1*-like, alpha-1-antitrypsin-like], *NME4* [NME/NM23 nucleoside diphosphate kinase 4], and *TAT* [tyrosine aminotransferase]) were shared between the bird types (Table S3). In mammals, *ANGPTL3* is in part involved in regulation of lipid and glucose metabolism by inhibiting the lipolysis of triglyceride-rich lipoproteins [29,30]. This transcript was highly down regulated in the AFB_1_-treated turkeys (log_2_FC = −7.94 and −14.13 in EW and DT, respectively). Similarly down regulated was *GC* an important protein in vitamin D transport and storage, actin-scavenging, and enhancement of complement component 5a activity for neutrophils in inflammation and during macrophage activation [31]. Comparison analysis in IPA found the most significant canonical pathways to include “Axonal Guidance Signaling” and “Hepatic Fibrosis/Stellate Cell activation”. Hepatic stellate cells are closely linked to the progression of hepatic fibrosis [32].

#### 2.2.1. Shared DE Genes

Changes in expression of the 3380 shared significant DE genes were highly correlated (r^2^ = 0.909, F = 0.010, Figure S1) and essentially linear, except for genes with the greatest down regulation where log_2_FC in the domestic birds tended to be of greater magnitude than observed for the wild birds. This is consistent with a common physiological response to AFB_1_ exposure. Of the 3380 shared DE genes, 1609 IDs mapped to the *G. gallus* gene REFLIST and statistical overrepresentation tests (PANTHER) of these shared DE genes found the greatest enrichment in the Biological Process category for “amino acid processes” and “negative regulators of hemostasis and wound healing” (Table S4). Comparison analysis in IPA found the most significant toxicology functions consistent with cellular damage. Categories with the greatest number of included genes included “liver hyperplasia/hyperproliferation” (*p* = 5.10 × 10^−41^), “cardiac hypertrophy” (*p* = 2.91 × 10^−12^), “renal necrosis/cell death” (*p* = 7.78 × 10^−12^), and “liver steatosis” (*p* = 1.75 × 10^−11^). Highest activation (Z) scores were obtained for “Integrin Signaling”, “Rho Family GTPase signaling”, and “NFAT regulation of immune response” pathways.

Only two loci among the 3380 shared significant DE genes showed opposite directional expression changes between wild and domesticated birds. *CD96*, a T cell-specific receptor, was significantly up regulated in response to AFB_1_ in the EW birds (log_2_FC = 3.83) but down regulated in DT (log_2_FC = −2.15). CD96 may play a role in the adhesive interactions of activated T and NK cells when actively engaging diseased cells within areas of inflammation [33]. A second locus (*LOC104911020*, serum amyloid A protein-like) was significantly down regulated in response to AFB_1_ in EW birds (log_2_FC = −3.99), but up regulated in DT (log_2_FC = 2.22). Serum amyloid A (SAA) proteins are a family of apolipoproteins produced primarily by the liver and are associated with high-density lipoprotein in plasma [34].

#### 2.2.2. Unique Responses

Although the greatest number of DEGs was shared between the domesticated and wild turkey comparisons, 796 DEGs were uniquely affected in the wild birds exposed to AFB_1_ in comparison to their controls (Figure 2). Up-regulated genes with the greatest fold change in the EW birds (Table S3) included several transcription factors (*DMRT2*, *FOXF2*, *HOXD10*, *HOXA9*, *HOXD8*) and transporters (*LOC100548321* [pendrin], *SLC13A1*, *SLC6A18*). DEGs with the greatest negative fold change (down regulated) include the cytochrome P450s (*LOC100548279* [*CYP2K4*-like], *LOC100546803* [*CYP8B1*], and *LOC100539035* [*CYP7A1*]), metabolic inhibitors (*LOC100542224* [alpha-1-antitrypsin-like], *INHBC* [inhibin, beta C], *LOC104912821* [ovostatin homolog], *LOC104915655* [alpha-2-macroglobulin-like]), and several ncRNAs. Of the 796 unique DEGs in the EW comparison, 336 had mapped IDs in *G. gallus* REFLIST and overrepresentation tests in PANTHER found greatest enrichment in the Biological Process category were genes in the GO classifications of “negative regulation of neurogenesis” (GO:0050768) and “negative regulation of cell development” (GO:0010721) with 5.58- and 5.13-fold enrichment, respectively (Table 3). Effected Cellular Component groups included “elements of the sarcolemma” (GO:0042383) and “proteinaceous extracellular matrix” (GO:0005578) enriched by 7.65- and 3.92-fold, respectively.

A greater number of DEGs (1241) were uniquely affected in the domesticated birds exposed to AFB_1_, in comparison to their controls. Of the 50 DEGs with the greatest fold change in the domesticated birds, only two were up regulated; *SMIM24* (small integral membrane protein 24) and *LOC100546964* (cis-aconitate decarboxylase-like) (Table S3). The function of SMIM24 is currently unknown. In humans, cis-aconitate decarboxylase (*ACOD1* = *IRG1*) is highly expressed in mammalian macrophages during inflammation where it catalyzes itaconic acid production [35]. Among the most down-regulated loci in DT were *ANGPTL3* (angiopoietin-like 3) which in humans, is expressed predominantly in the liver and functions in angiogenesis, and Fibrinogen (*FGA*, *FGB*, and *FGG*) and other coagulation components like coagulation factor IX (*F9*). This set of down-regulated DEGs also included *LOC100547030* (a cytochrome P450 2W1-like gene). In humans, CYP2W1 is able to metabolically activate several pro-carcinogens, including AFB_1_, into cytotoxic products [36]. Due to its selective expression, *CYP2W1* is suggested as a potential prognostic biomarker in hepatocellular and other carcinomas [37].

GO analysis of the DEGs unique to the domestic turkey liver indicate a number of distinctive responses to AFB_1_. Of the 1241 DEGs, 636 mapped to IDs in *G. gallus* REFLIST and overrepresentation tests in PANTHER found the greatest enrichment for biological process categories “organelle fission” (GO:0048285), “oxidation-reduction process” (GO:0055114), and “regulation of immune system process” (GO:0002682) (Table 4). Cellular component categories were enriched for mitochondrial and membrane components.

### 2.3. Differential Transcriptomic Expression: Eastern Wild vs. Domesticated Turkey

#### 2.3.1. Control

**Figure 3:**
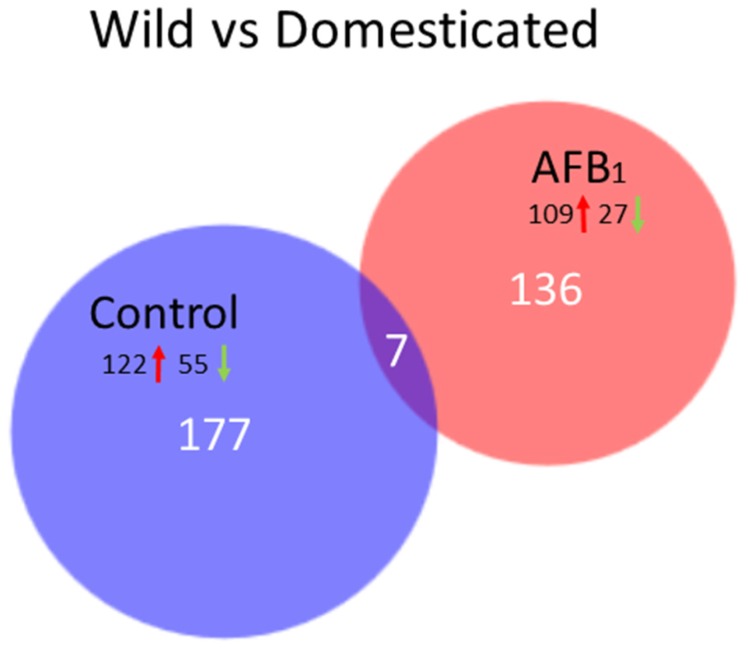
Distribution of differentially expressed of liver genes between turkey types (Wild and Domesticated). For each comparison, the number of genes with FDR *p*-value < 0.05 and |log_2_FC| > 2.0 shared or unique to each treatment group are indicated. Circle size is proportional to the number of genes and direction of expression change (↑ or ↓) is given for the genes in each group.

Comparison of the transcriptomes of control EW and DT birds found 774 DEGs (FDR *p*-value < 0.05, log_2_FC = −8.826 to 8.213), with 184 having log_2_FC > 2.0 (Figure 3, Table S5). Of the 184 genes, seven were shared in common in the EW vs. DT AFB_1_ comparisons (Figure 3). The shared loci included 5 genes up regulated in EW birds (*ANGPTL3*, *CAMK4*, *LOC100538933* [probable ATP-dependent RNA helicase *DDX60*], *LOC100545362* [ncRNA], *LOC104912934* [ncRNA]), and two that were down regulated (*LOC104910139* [ncRNA], *LOC104915640* [*KIAA1755* homolog]) when compared to DT. These shared genes are primarily metabolic and transcriptional regulators. In mammals, ANGPTL3 [angiopoietin-like 3] is a hepatokine involved in regulation of lipid and glucose metabolism and in the regulation of angiogenesis [37], CAMK4 [calcium/calmodulin-dependent protein kinase IV] is implicated in transcriptional regulation in immune and inflammatory responses [39], and DDX60 positively regulates DDX58/RIG-I- and IFIH1/MDA5-dependent type I interferon and interferon inducible gene expression [40].

**Figure 4:**
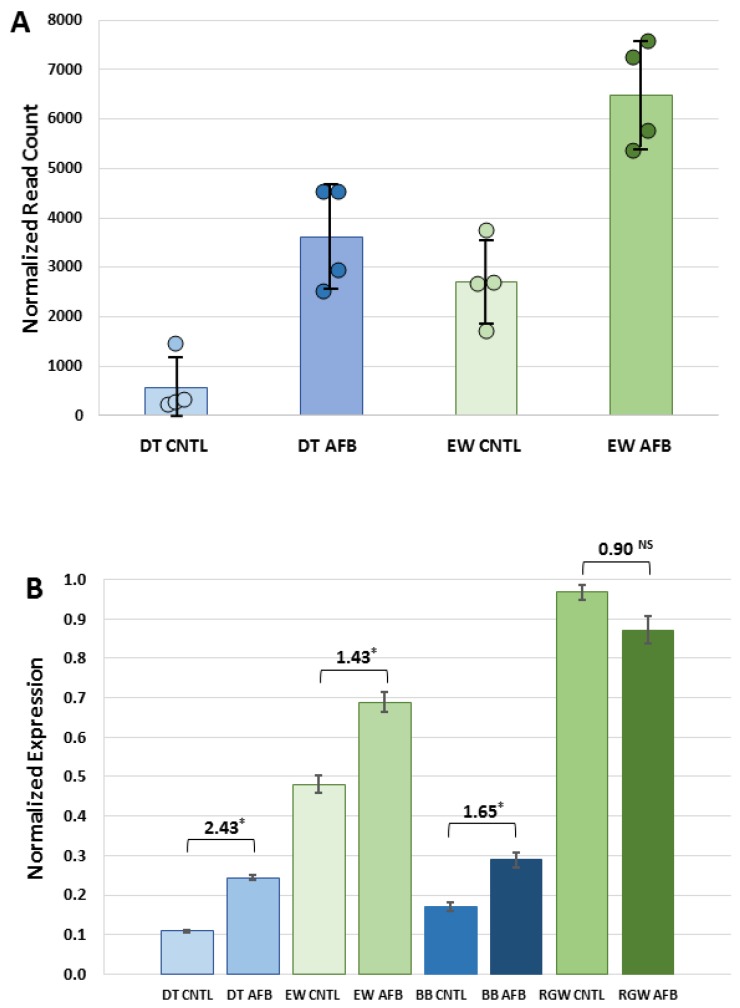
Effect of AFB_1_ on expression of *GSTA3* in the livers of turkeys. (**A**) Mean normalized RNA-seq read counts. For each treatment group, individual read counts are indicated by closed circles. Error bars denote standard deviation of the mean. (**B**) Relative expression as measured by qRT-PCR. For each group the fold change (ΔΔCt) between AFB_1_-treated and control birds is given. Asterisks denote significant comparisons (*p*-value < 0.01).

Of the 177 DEGs unique to the control group birds the majority (69%) were up regulated in the wild birds as compared to the domesticated birds. GO analysis found only a single biological process category (“single-organism process” GO:0044699) enriched among these genes (fold enrichment = 1.66, *p*-value = 4.15 × 10^−2^). Interestingly, the up-regulated DEGs observed in the control-diet comparison also included glutathione S-transferase A3 (*GSTA3*). This gene was expressed at a 5.3-fold higher level (log_2_FC = 2.313) in the EW birds when compared to the DT birds (Figure 4A), suggesting a higher constitutive expression in the former.

The genes showing the greatest positive fold change was the Interferon-inducible iron-sulfur cluster-binding antiviral protein *RSAD2*, radical S-adenosyl methionine domain containing 2, with log_2_FC = 8.2 and the interferon alpha-inducible protein 27-like 2B (*IFI27L2B*, *LOC100550948*). In mammals, RSAD2 can inhibit a wide range of DNA and RNA viruses, and has also been shown to play a role in CD4+ T-cells activation and differentiation [41], whereas IFI27L2B mediates virus-induced apoptosis [42]. These suggest heightened immune system activity in the wild birds.

Two genes involved in cell cycle control and chromosomal replication were also expressed at a significantly higher level in the wild bird controls. *CD1*, chromatin licensing and DNA replication factor 1 and *MCM3*, minichromosome maintenance complex component 3 had log_2_FC = 2.74 and 2.4, respectively (Table S5). Interestingly several other genes involved “cell cycle control” and “chromosomal replication” were also significantly up regulated in the wild birds, although with log_2_FC < 2.0 (Table S2). These included *CDC45* (cell division cycle 45), *DNA2* (DNA replication helicase/nuclease 2), the minichromosome maintenance complex components *MCM2*, *MCM4*, *MCM5*, *MCM6*, and *MCM8*, and *POLE* (DNA polymerase epsilon, catalytic subunit). These together with *CD1* and *MCM2* were components of the most significant Canonical Pathway identified by IPA (*p* = 1.1 × 10^−8^, ratio 0.25). Genes showing the greatest negative fold change were *GYG2* (glycogenin 2), *IQCD* (IQ Motif Containing Protein D), and *LOC100540418* (BPI fold-containing family C protein-like) (Table S5). GYG2 is involved in the initiation reactions of glycogen biosynthesis [43], and IQCD has been shown to interact with the RXR nuclear hormone receptor, and is thought to function as a transcriptional coactivator [44].

#### 2.3.2. AFB_1_ Treatment

Comparison of the transcriptomes of EW and DT birds fed the AFB_1_ diet revealed 903 DEGs (FDR *p*-value < 0.05, log_2_FC = −7.987 to 9.262), of which, 143 had log_2_FC > 2.0 (Figure 3, Table S5). Of these 143 DEGs, 136 were unique to the AFB_1_-treated birds. The majority (76%) of the DEGs were up regulated in EW relative to DT in a similar fashion to that seen for the control-group comparison. Unlike the control-fed groups, GO analysis found the DE genes from the AFB_1_ between-line comparison highly enriched in several biological process categories (Table 5) including processes related to coagulation, inflammatory response and apoptosis. For example, up regulated in EW relative to DT were three components of the blood clotting factor fibrinogen (*FGA*, *FGB*, and *FGG*) and several other coagulation-related genes (vitamin K-dependent coagulation factor IX (*F9*), serpin peptidase inhibitor, member 10 (*SERPINA10*), histidine-rich glycoprotein (*HRG*), serpin peptidase inhibitor, clade C (antithrombin), member 1 (*SERPINC1*)). Although down-regulated genes did not show significant enrichment for a particular bioprocess, EW birds may produce less of the pro-inflammatory cytokine IL-17A (*LOC100546746*).

Expression of many enzymes responsible for xenobiotic metabolism, with an emphasis on those with specificity towards AFB_1_, was significantly altered by the AFB_1_ treatment (Table 6). With a few exceptions, AFB_1_ largely caused down regulation of these genes in both types of turkeys when compared to controls; within the control groups, the number of DEGs was considerably smaller and the magnitude of expression changes was also smaller, but directionally similar. Hepatic expression of CYP and GST genes was typically greater in EW vs. DT. Five cytochrome P450 loci; *CYP1A5* (cytochrome P450, family 1, subfamily A, polypeptide 5, log_2_FC = 2.404), *LOC100547030* (cytochrome P450 2W1-like, log_2_FC = 5.87), *LOC100542486* (cytochrome P450 1A4, log_2_FC = 3.150), *LOC100548433* (cytochrome P450 2K1-like, log_2_FC = 3.025), and *LOC104915479* (cytochrome P450 2H1-like, log_2_FC = 2.135) were among the unique DEGs in the EW vs. DT comparison.

The turkey possesses six α-GST cluster genes, all of which possess detectable enzymatic activities toward prototype substrates in a recombinant expression system [19], unlike hepatic forms. Expression of the GSTAs was significantly altered by AFB_1_ treatment. With the exception of *GSTA3*, *GSTA1.1*, *1.3*, *2* and *4* were down regulated, while *GSTA3* increased with dietary AFB_1_ in DT but not EW (log_2_FC = 1.4667, Table 6). It is noteworthy that *GSTA3* expression was significantly higher in EW birds when compared to DT birds for both control (log_2_FC = 2.3130) and AFB_1_ (log_2_FC = 0.9211) group comparisons (Table 6, Figure 4A).

Expression differences in *GSTA3* observed in RNA-seq read counts were confirmed by qRT-PCR. *GSTA3* expression varied widely among treatment groups with experiment-wise threshold values (ΔCt) ranging from 17.27 to 26.06. Expression of *GSTA3* transcripts was significantly higher in AFB_1_-treated birds than controls for both genetic groups (EW, *p* = 0.0061 and DT, *p* = 0.0036). Relative *GSTA3* expression was also similarly variable in the other commercial (BB) and wild-type birds (RGW) (Figure 4B) where *GSTA3* expression was higher in AFB_1_-treated birds. This difference, however, was only significant in the BB comparison (*p* = 0.0015). Relative expression in BB birds was slightly higher than observed in the DT (Nicholas strain) birds and also higher in RGW birds (Rio Grande wild) when compared to EW. This result demonstrates that the differences observed in *GSTA3* expression between the EW and DT birds is not unique to these genetic lines but is a broader, wild versus domesticated-bird phenomenon.

## 3. Discussion

When compared to their domestic relatives, wild turkeys are relatively resistant to aflatoxicosis. This difference is largely due to functional hepatic GSTA-mediated detoxification activity of the bioactive electrophilic AFBO intermediate that is completely lacking in domesticated birds [20]. The present data indicates other pathways may also account for difference in AFB_1_ susceptibility, such as cellular regulation, modulation of apoptosis, inflammatory responses, and other pathways relevant to AFB_1_ pathogenesis. The liver is the principal organ of AFB_1_ bioactivation and detoxification [6,21,22,24,45]. In turkeys, AFB_1_ causes reduced feed intake, weight gain, and immunological function in a dose-dependent fashion [46,47]. Dietary exposure in poultry causes lipid accumulation, resulting in hepatomegaly and increases in liver:body weight ratios [48,49,50]. During the 14 day exposure, decreased relative liver mass initially occurred in both EW and DT consistent with that observed in chickens [49] and wild turkeys [9].

Numerous significant DEGs occurring in the livers of AFB_1_-treated birds have potential roles in lipid metabolism or accumulation. AFB_1_ is known to alter lipid metabolism and increase lipid content resulting in pale or yellowed pigmentation [46]. Dietary AFB_1_ primarily down regulated several hepatic apolipoprotein genes (cofactors in lipid binding and transport) in the turkey, and dis-regulation of genes, such as *ANGTPL3*, would have direct effects on lipids. Significant up regulation of *ANGTPL3* was observed for both EW and DT birds treated with AFB_1_. This would likely stimulate synthesis of plasma triglycerides (TG) via the inhibition of lipoprotein lipase (LPL) activity. In both AFB_1_-treated groups, *LPL* was significantly down regulated (log_2_FC = −2.905 and −6.032 in EW and DT birds, respectively). LPL functions in the hydrolysis of triglycerides in lipoproteins and is essential to lipid metabolism and storage. Significant down regulation of *LPL* was also observed in our previous analyses of AFB_1_-treated domesticated Orlopp turkeys [26] and decreased expression of LPL occurs in AFB_1_-treated chickens [50].

As expected, the significant hepatic DEGs included the Phase I and II detoxifying enzymes that we have shown are relevant to AFB_1_ exposure in turkeys (Table 6). Previous studies have demonstrated efficient epoxidation by hepatic turkey cytochromes CYP1A5 and CYP3A37 [16]. At environmentally-relevant hepatic concentrations (<50 uM) CYP1A5 bioactivates the majority (~98%) of AFB_1_ [17,21], whereas CYP3A37 predominates at much higher substrate concentrations unlikely to be achieved in the livers of exposed animals [16]. Based on RNA-seq, it is clear that dietary AFB_1_ significantly down regulated *CYP1A5* in both EW and DT birds, but more significantly so in DT. This result is at odds with our earlier findings in another strain of DT (Orlopp) where almost no expression change was observed for *CYP1A5* and *CYP3A37*, and where none of the transcripts associated with CYP genes had significant DE as a result of AFB_1_ treatment [26]. Significant down regulation of *CYP1A5* in response to AFB_1_ was also observed in ducks, another avian species with high AFB_1_ susceptibility [51]. Several other P450 genes in addition to *1A5* and *3A37* had significant DE in the present study (Table 6), including both *CYP2W1* and *CYP2K1*. Interestingly, these genes have been shown in other species to activate AFB_1_ into cytotoxic products [52,53]. We have found *CYP2W1*-like transcripts to have significant DE in DT embryos challenged with AFB_1_ [28]. Down regulation of *CYP1A5* in both EW and DT birds could affect their overall ability to bioactivate AFB_1_. However, as this expression change was seen in both bird types, it does not account for the differences seen in AFB_1_ susceptibility [16].

Expression of GSTs with affinity toward AFBO is a known predictor of relative AFB_1_ resistance [20]. Constitutive expression of *GSTA3*, the ortholog to the putative AFB_1_-protective GSTA3 isoform in mice [18] was significantly higher in EW than in DT birds. Dietary AFB_1_ caused significant down-regulation of hepatic α-class GSTs, with the exception of *GSTA3*, where increased expression of this isoform was observed in the AFB_1_-treated DT group. This pattern was also observed in the qRT experiments of other wild (RGW) and domesticated (BB) turkeys. A similar pattern of *GSTA3* expression in response to AFB_1_ was also observed in turkey embryos early after exposure, where small increases were observed in DT [28] and in ducks [51].

Expression of *GSTA3* mRNA in turkeys is not correlated with AFB_1_ sensitivity in that domesticated birds lack hepatic GST-mediated AFBO conjugating activity [19], despite expression of *GSTA3*. Hepatic cytosols isolated from wild turkeys possess functional AFBO-trapping GSTs [20]. While hepatic GSTs in DT lack detoxification activity, with or without AFB_1_ treatment, increased *GSTA3* expression in DT in response to AFB_1_ may reflect a greater inflammatory response or perhaps an indicator of hepatocyte injury. Although GSTs are toxicologically important for their role in “trapping” electrophilic intermediates by conjugating with the nucleophilic GSH, they may also play a role in cell signaling through binding of non-substrate ligands to mediate cell proliferation and cell death [54]. Up regulation of *GSTA*s may also reflect antioxidant functions as AFB_1_, exposure in poultry can lead to oxidative stress and lipid peroxidation [55,56]. When combined, these results support the hypothesis that the greater ability of wild turkeys to detoxify AFB_1_ is related to higher constitutive expression of *GSTA3*, coupled with an inherited (genetic) difference in functional expression in domesticated birds. Expression in these *CYP* and *GSTAs* suggests that the physiological response to AFB_1_ is mediated through genes not experimentally linked in the turkey to AFB_1_ metabolism.

Up regulation of transcription factors and metabolic inhibitors characterized the shared response to AFB_1_. Taken together, these are genes that comprise the molecular mechanisms underlying aflatoxicosis. Recurrent themes amongst the many DEGs of AFB_1_-treated birds are linked by functional analysis to inflammation, apoptosis, the cell cycle (cancer), or lipid regulation, suggesting common underlying regulation. For example, recent studies of AFB_1_-induced hepatocellular carcinoma have examined regulatory ncRNAs (miRNA and lncRNA) [57,58]. Studies in the rat, another AFB_1_-susceptible species, have found coincident DE of transcripts that are related to these same functions and specific lncRNAs in hepatocellular carcinomas [59,60]. Our study of miRNA expression in the same turkey liver tissues used in the present study is currently underway (Coulombe, unpublished).

Transcriptome analysis not only includes genes responding to the presence of AFB_1_, but also reveals genes dis-regulated as a response to toxic insult. Significant up regulation was seen for several vasoactive peptides, including, neuropeptide Y (*NPY*), somatostatin (*SST*), substance P (tachykinin, *TAC1*), and vasoactive intestinal peptide (*VIP*), suggesting altered sinusoidal blood flow with AFB_1_ treatment. Also, affected were extracellular matrix proteins including glycoproteins (e.g., HAPLN1 and HAPLN3), protein receptors (*KERA*, *LAMB3*, *LUM*, *LRRN2*, and *LRRN3*), proteinase inhibitors (*TIMP4*), signaling molecules (*SFRP1*, *Wnt6* and *7a*), and structural proteins (*COL10A1*, *FRAS1*). Expression of the majority of the ADAM metallopeptidases was altered in AFB_1_ treatment (Table S2). Some of these proteases are thought to be involved in regulating matrix degradation [61]. Unique response in the EW birds was seen in genes that negatively regulate cellular processes, components of the extracellular matrix and accumulation of coagulation factors. DT birds showed greater up regulation of genes responding to inflammation, which was likely due to the reduced ability to detoxify AFBO. Dis-regulation of extracellular matrix proteins is a resulting effect of chronic liver injury [32]. Aflatoxin inhibits cell-mediated immunity in domestic poults [47,62] with the suppression of lymophoblastogenesis [9], T-helper, or cytotoxic T-cell activity [63].

Multiple genes involved in pathways of coagulation (*FGA*, *FGB*, *FGG*, *F9*, *HRG*, *SERPINA10*, and *SERPINC1*) were expressed at higher levels in EW as compared to DT, where they were among the genes with the highest negative fold change. Lower expression of coagulation factors was also seen in livers of domesticated turkey embryos after just 5 days of exposure to AFB_1_ [28]. AFB_1_ has been shown to increase blood clotting times in poultry [64,65] and activities of coagulation factors, such as F9, were reduced by dietary AFB_1_ in chickens [50,65]. Effects on hemostasis are more dramatic in turkeys than chickens [66]. In comparison, only small non-significant increases in prothrombin times were seen in wild turkeys exposed to AFB_1_ [9], which is consistent with the gene expression patterns observed in the liver transcriptomes.

In a previous comparison of EW and DT after *in ovo* exposure [28], we used RNA-seq to examine gene expression responses to AFB_1_ in the embryonic hepatic transcriptomes and identified gene expression effects dependent on exposure time and turkey type. Most notable in turkey embryos was the more rapid response of the DT, which was likely due to their lack of GST activity towards the AFBO-epoxide. The present study was designed to contrast gene expression responses in the hepatic transcriptome of growing domesticated and wild turkeys during AFB_1_ exposure. In conclusion, our findings emphasize the differential response of these genetically distinct birds, demonstrating significant differences in expression of Phase I and Phase II genes and in genes important in cellular regulation, modulation of apoptosis, and inflammatory responses. The molecular basis for the differences in AFB_1_ detoxification observed between EW and DT birds, and the mechanism of GSTA silencing in DT remain under investigation.

## 4. Materials and Methods

This study used two turkey subspecies previously demonstrated to vary in AFB_1_-detoxifying GST activity. Eggs from domesticated (DT = Nicholas) and a wild subspecies (Eastern wild = EW, *Meleagris gallopavo silvestris*) were obtained from Privett Hatchery (Portales, NM, USA) and hatched at Utah State University. Birds were sexed by PCR [67]. Male turkey poults were maintained on an *ad libitum* standard grow-up soy-based diet and acclimated to the facility for two weeks. At the end of this period, males from each line (*n* = 8 for EW and *n* = 10 for DT) were equally assigned to one of two treatment groups and subjected to a short-term AFB_1_-treatment protocol [21,68]. For the AFB_1_ treatment, the diet of challenge birds was amended beginning on day 15 with 320 ppb AFB_1_ (Sigma-Aldrich, Inc., St. Louis, MO, USA) that continued for 14 days. Control birds continued on the standard diet with AFB_1_ levels below detection limits (<10 ppb), based on testing of 50 g of feed extracted and cleaned using Mycosep 112 AflaZON cleanup columns (Romer Labs., Union, MO, USA), and examined by HPLC. Birds were weighed three times per week and feed and water availability checked daily. At the conclusion of the 14 day challenge period, birds were sacrificed by CO_2_ asphyxiation and blood collected by cardiac puncture for DNA and serological analysis. Livers were removed, examined, weighed, sampled, and fixed in neutral buffered formalin for histological examination. Portions of the liver tissues infused with RNAlater (ThermoFisher Scientific, Waltham, MA, USA) for RNA-Seq analysis. All of the procedures were under the authority and institutional approval of Utah State University’s Animal Use and Care Committee. Ethical approval code: 2670, Date of approval: 26 September 2016.

### 4.1. RNA Isolation and Sequencing

Total RNA was isolated from each sample by TRIzol extraction (Ambion, Inc., Foster City, CA), DNase-treated (Turbo DNA-freeTM Kit, Ambion, Inc.), and stored at −80 °C. Initial RNA concentration and quality was assessed by spectrophotometry (Nanodrop 8000). RNA samples were submitted for library preparation and sequencing at the University of Minnesota Genomics Center (UMGC). Replicate samples were sequenced from each treatment group (*n* = 4). Each sample was quantified by RiboGreen assay (Invitrogen Corp., Carlsbad, CA, USA) and RNA integrity confirmed on a 2100 Bioanalyzer (Aligent Technologies, Santa Clara, CA, USA). Each sample had clear 18S and 28S peak separation on the electropherograms and an average RNA Integrity Number (RIN) of 6.3. Indexed libraries (*n* = 16) were constructed with 1 μg of total RNA/sample with the TruSeq RNA Sample Preparation Kit version 2 (Illumina, Inc., San Diego, CA, USA) and size selected for approximately 200 bp inserts. Libraries were multiplexed, pooled, and sequenced over two lanes on the HiSeq 2000 using v3 chemistry (Illumina, Inc.) to produce 101-bp paired-end reads. Data are deposited in the NCBI Gene Expression Omnibus (GEO) repository as part of SRA BioProject 346253.

### 4.2. RNA-seq Data Analyses

Sequence reads were groomed (Trimmomatic, [69]) and quality checked (FastQC, [70]) prior to read mapping (Bowtie v2.2.4.0) on the turkey genome (UMD 5.0, NCBI Annotation 101). Read counts were normalized in CLC Genomics Workbench (CLCGWB v. 8.0.2, CLC Bio, Aarhus, Denmark) by dividing the total read counts by the group sample sum with the results being expressed as reads per 12.2 M. Hierarchical clustering of samples was performed (based on Euclidean sample distances with single linkage) in CLCGWB. Principal component analysis (PCA), Volcano plots, and Venn diagrams were used to visualize expression data and the results of significance testing. Empirical analysis of differential gene expression and ANOVA were performed in CLCGWB on EdgeR-normalized read counts. Pair-wise comparisons between treatment groups were made in CLCGWB following the standard workflow Wald test with multi-comparison *p*-values < 0.05 being considered as significant (Bonferroni and FDR corrected). In each pair-wise comparison, significant DE genes were used to investigate affected gene pathways using Ingenuity Pathway Analysis (IPA, Ingenuity Systems, Redwood City, CA, USA). Gene enrichment tests were performed using the PANTHER Overrepresentation Test (GO Consortium release 20150430, [38]).

### 4.3. Quantitative Real-Time PCR

To more broadly examine the expression profile response of *GSTA3* to dietary AFB_1_, quantitative real-time PCR (qRT-PCR) was performed on both domesticated and wild turkey liver samples. Samples included AFB_1_-treated and control animals (six per group) from the domesticated Nicholas turkey (DT) and Eastern Wild (EW) experiment, plus AFB_1_-treated and control animals (six per group) of a parallel experiment that included domesticated Broad Breasted White (BB), and birds of the Rio Grande subspecies (RGW, *M. g. intermedia*) of wild turkey. Four of the six samples for the DT and EW groups were in common with the RNA-seq study.

Briefly, cDNA was synthesized from DNase-treated liver mRNA (TRIzol extracted) using Invitrogen Super Script IV First-strand synthesis kit (Invitrogen, Carlsbad, CA, USA). Expression analysis of gene-specific amplicons was performed with the iTaq Universal SYBR Green Supermix (BioRad, Hercules, CA, SA) with the CFX96 touch real time detection system (BioRad, Hercules, CA, USA). Primers were designed within Primer3 software (http://www.ncbi.nlm.nih.gov/tools/primer-blast) from accessioned genomic DNA sequence (NM_001303157.1) to span an exon/exon junction and at least one intron in the amplicon. RefFinder software was utilized to determine the most stable reference gene. Several normalizing genes were tested for uniformity between treatments and lines and RNA polymerase II subunit D (*POLR2D*, XM_003208947) was found to have the highest stability value (0.848). Target gene reactions were conducted in triplicate, and *POLR2D*, no template and gDNA controls were run in duplicate. All of the reactions included a disassociation curve to confirm a single product and to preclude the possibility of dimers amplifying. Expression in each RNA sample was normalized first to the control gene *POLR2D*. Results were interpreted using the Double Delta Ct Analysis (ΔΔCt, [71]) and a comparative Ct approach. Expression analysis was performed within the Biorad CFX Maestro software package following the standard ΔΔCt workflow.

## Figures and Tables

**Table 1:** Summary of RNA-seq data for turkey liver transcriptomes.

Line	Group	Replicate	PE Reads	Median Read Quality R1	Median Read Quality R2	% Mapped	% Concordant	Estimated Insert Mean (bp)	Observed Genes	Expressed Genes	% Genes Expressed
**Eastern Wild**	**CNTL**	EW9L	12,148,654	36.5	36.6	91.7	86.8	172	15,857	14,804	70.5
		EW10L	14,641,781	36.4	36.5	90.9	85.7	173	16,483	14,833	70.6
		EW12L	11,740,806	36.9	36.8	91.1	85.8	191	16,506	15,448	73.5
		EW13L	12,466,171	36.9	36.9	90.9	85.4	191	16,572	14,935	71.1
		**Mean**	12,749,353.0	36.68	36.70	91.15	85.93	181.8	16,354.5	15,005.0	71.4
	**AFB**	EW1L	11,719,049	36.9	37.0	89.7	83.6	192	17,914	17,073	81.3
		EW2L	12,615,195	36.9	37.0	90.1	84.2	191	17,911	16,523	78.6
		EW3L	12,625,962	36.9	36.9	90.5	84.7	192	18,026	17,194	81.8
		EW4L	12,468,136	36.9	36.9	89.9	84.0	193	18,054	17,230	82.0
		**Mean**	12,357,085.5	36.90	36.95	90.05	84.13	192.0	17,976.3	17,005.0	80.9
**Domesticated**	**CNTL**	N11L	12,448,496	36.7	36.8	92.4	87.7	172	15,993	14,837	70.6
		N12L	12,857,795	36.7	36.7	92.3	87.7	172	16,136	14,399	68.5
		N13L	11,417,338	37.2	37.4	91.4	85.7	212	16,634	15,584	74.2
		N14L	12,099,335	36.7	36.6	91.9	87.0	170	15,952	14,780	70.4
		**Mean**	12,205,741.0	36.83	36.88	92.00	87.03	181.5	16,178.7	14,900.0	70.9
	**AFB**	N1L	11,388,753	37.2	37.4	90.3	84.3	212	17,967	17,109	81.4
		N2L	12,827,964	37.1	37.3	89.8	83.6	213	18,083	16,752	79.7
		N3L	10,821,683	37.1	37.3	90.4	84.4	211	17,903	17,048	81.1
		N4L	11,144,371	37.2	37.3	90.1	84.0	211	17,989	17,119	81.5
		**Mean**	11,545,693.0	37.15	37.33	90.15	84.08	211.8	17,985.5	17,007.0	81.0
**Mean**			12,214,468.06	36.89	36.96	90.84	85.29	191.75	17,123.75	15,979.25	76.1

For each library the total number of concatenated reads, median read qualities (R1 and R2), estimated mean insert length (bp), number of and percentage of aligned reads, percentage of concordant reads, and the number and percentage of observed genes (mapped reads > 1) and expressed genes (mean group normalized read count > 3.0) are given.

**Table 2:** Summary of genes with significant differential expression (DE) in pair-wise comparisons of treatment groups.

Comparison	Groups	Expressed Genes	Shared Genes	Unique Genes/Group	FDR Pval < 0.05	|log_2_FC| > 1.0	|log_2_FC| > 2.0
AFB_1_ effect	EW (AFB vs. CNTL)	17,342	14,908	2147/287	9620	7168	4176
	DT (AFB vs. CNTL)	17,403	14,669	2407/328	11,325	8001	4621
Line	CNTL (EW vs. DT)	15,525	14,667	528/330	744	495	184
	AFB (EW vs. DT)	17,411	16,719	336/356	903	456	143

For each comparison, the treatment groups, number of genes with significant FDR *p*-value, and the numbers of significant genes that also had |log_2_ fold change| > 1.0 and > 2.0 are given.

**Table 3:** Summary of PANTHER Overrepresentation Test of the 796 unique differentially expressed (DE) genes in livers of Eastern wild turkeys compared to controls after AFB_1_ exposure.

Category	*Gallus gallus*—REFLIST Genes (15,789)	Observed Turkey Genes	Expected	over/under	Fold Enrichment	*p*-Value
**GO biological process complete**						
negative regulation of neurogenesis (GO:0050768)	101	12	2.15	+	5.58	1.28 × 10^−2^
negative regulation of cell development (GO:0010721)	119	13	2.53	+	5.13	1.21 × 10^−2^
negative regulation of nervous system development (GO:0051961)	111	12	2.36	+	5.08	3.29 × 10^−2^
regulation of system process (GO:0044057)	184	16	3.92	+	4.09	1.58 × 10^−2^
regulation of membrane potential (GO:0042391)	185	16	3.94	+	4.06	1.69 × 10^−2^
regulation of neuron differentiation (GO:0045664)	233	19	4.96	+	3.83	4.83 × 10^−3^
regulation of neurogenesis (GO:0050767)	279	20	5.94	+	3.37	1.72 × 10^−2^
regulation of nervous system development (GO:0051960)	323	23	6.87	+	3.35	3.53 × 10^−3^
neurological system process (GO:0050877)	337	22	7.17	+	3.07	2.45 × 10^−2^
system process (GO:0003008)	559	30	11.9	+	2.52	2.36 × 10^−2^
generation of neurons (GO:0048699)	589	31	12.53	+	2.47	2.40 × 10^−2^
neurogenesis (GO:0022008)	636	33	13.53	+	2.44	1.57 × 10^−2^
nervous system development (GO:0007399)	886	44	18.85	+	2.33	1.03 × 10^−3^
regulation of multicellular organismal process (GO:0051239)	1083	52	23.05	+	2.26	2.01 × 10^−4^
system development (GO:0048731)	1613	64	34.33	+	1.86	4.44 × 10^−3^
cell communication (GO:0007154)	2250	85	47.88	+	1.78	3.50 × 10^−4^
single organism signaling (GO:0044700)	2201	83	46.84	+	1.77	5.71 × 10^−4^
multicellular organismal process (GO:0032501)	2417	91	51.44	+	1.77	1.16 × 10^−4^
signaling (GO:0023052)	2205	83	46.92	+	1.77	6.18 × 10^−4^
multicellular organism development (GO:0007275)	1814	68	38.6	+	1.76	1.37 × 10^−2^
single-multicellular organism process (GO:0044707)	2120	79	45.11	+	1.75	2.09 × 10^−3^
anatomical structure development (GO:0048856)	1971	71	41.94	+	1.69	3.16 × 10^−2^
single-organism process (GO:0044699)	6098	180	129.77	+	1.39	1.05 × 10^−4^
Unclassified (UNCLASSIFIED)	6357	84	135.28	-	0.62	0.00 × 10
**GO cellular component complete**						
sarcolemma (GO:0042383)	43	7	0.92	+	7.65	4.23 × 10^−2^
proteinaceous extracellular matrix (GO:0005578)	168	14	3.58	+	3.92	1.85 × 10^−2^
extracellular matrix (GO:0031012)	249	18	5.3	+	3.4	8.51 × 10^−3^
plasma membrane part (GO:0044459)	987	45	21.0	+	2.14	1.33 × 10^−3^
intrinsic component of membrane (GO:0031224)	3244	132	69.03	+	1.91	3.19 × 10^−12^
integral component of membrane (GO:0016021)	3200	129	68.1	+	1.89	1.70 × 10^−11^
plasma membrane (GO:0005886)	1834	71	39.03	+	1.82	4.27 × 10^−4^
membrane part (GO:0044425)	3689	142	78.5	+	1.81	1.39 × 10^−11^
cell periphery (GO:0071944)	1888	72	40.18	+	1.79	6.08 × 10^−4^
extracellular region part (GO:0044421)	1555	57	33.09	+	1.72	3.49 × 10^−2^
membrane (GO:0016020)	4745	154	100.98	+	1.53	7.69 × 10^−7^
organelle part (GO:0044422)	3580	45	76.18	-	0.59	1.11 × 10^−2^
intracellular organelle part (GO:0044446)	3472	41	73.89	-	0.55	2.59 × 10^−3^
nucleus (GO:0005634)	3106	35	66.1	-	0.53	3.12 × 10^−3^
organelle lumen (GO:0043233)	1703	15	36.24	-	0.41	2.45 × 10^−2^
intracellular organelle lumen (GO:0070013)	1703	15	36.24	-	0.41	2.45 × 10^−2^
membrane-enclosed lumen (GO:0031974)	1703	15	36.24	-	0.41	2.45 × 10^−2^
nuclear part (GO:0044428)	1764	15	37.54	-	0.4	1.01 × 10^−2^
Unclassified (UNCLASSIFIED)	6062	78	129.0	-	0.6	0.00 × 10
**GO molecular function complete**						
transporter activity	861	39	18.32	+	2.13	1.71 × 10^−2^
nucleic acid binding	1988	15	42.31	-	0.35	6.66 × 10^−4^
Unclassified	6653	112	141.58	-	0.79	0.00 × 10

Included categories had fold enrichment (number of DE genes divided by expected (Exp)) > 2.0. For each Gene Ontology category, the number of genes in the reference list and those differentially expressed in the turkey are given. DE turkey genes were matched to the chicken gene reference list for analysis in PANTHER [38]. *p*-values are as determined by the binomial statistic.

**Table 4:** Summary of PANTHER Overrepresentation Test of the 1241 unique differentially expressed (DE) genes in liver of domesticated turkeys after AFB_1_ exposure as compared to controls.

Category	*Gallus gallus*—REFLIST Genes (15789)	Observed Turkey Genes	Expected	over/under	Fold Enrichment	*p*-Value
**GO biological process complete**						
organelle fission (GO:0048285)	135	17	4.52	+	3.76	2.53 × 10^−2^
oxidation-reduction process (GO:0055114)	549	46	18.39	+	2.50	1.11 × 10^−4^
regulation of immune system process (GO:0002682)	408	33	13.67	+	2.41	2.40 × 10^−2^
signal transduction (GO:0007165)	2057	114	68.92	+	1.65	2.19 × 10^−4^
Signaling (GO:0023052)	2205	122	73.88	+	1.65	6.80 × 10^−5^
single organism signaling (GO:0044700)	2201	121	73.74	+	1.64	1.15 × 10^−4^
single-organism metabolic process (GO:0044710)	1745	95	58.47	+	1.62	8.40 × 10^−3^
cell communication (GO:0007154)	2250	122	75.38	+	1.62	2.13 × 10^−4^
cellular response to stimulus (GO:0051716)	2657	135	89.02	+	1.52	1.45 × 10^−3^
single-organism process (GO:0044699)	6098	309	204.31	+	1.51	1.54 × 10^−16^
response to stimulus (GO:0050896)	3224	159	108.02	+	1.47	5.26 × 10^−4^
single-organism cellular process (GO:0044763)	4263	205	142.83	+	1.44	1.46 × 10^−5^
regulation of cellular process (GO:0050794)	4810	220	161.16	+	1.37	2.08 × 10^−4^
regulation of biological process GO:0050789)	5131	227	171.91	+	1.32	1.83 × 10^−3^
biological regulation (GO:0065007)	5521	244	184.98	+	1.32	4.20 × 10^−4^
cellular process (GO:0009987)	7267	307	243.48	+	1.26	1.04 × 10^−4^
Unclassified	6357	130	212.99	-	0.61	0.00 × 10
**GO cellular component complete**						
mitochondrial inner membrane (GO:0005743)	213	22	7.14	+	3.08	4.57 × 10^−3^
mitochondrial membrane (GO:0031966)	273	26	9.15	+	2.84	2.75 × 10^−3^
mitochondrial envelope (GO:0005740)	290	27	9.72	+	2.78	2.69 × 10^−3^
organelle inner membrane (GO:0019866)	239	22	8.01	+	2.75	2.60 × 10^−2^
mitochondrial part (GO:0044429)	395	30	13.23	+	2.27	3.65 × 10^−2^
mitochondrion (GO:0005739)	819	50	27.44	+	1.82	3.85 × 10^−2^
membrane part (GO:0044425)	3689	193	123.6	+	1.56	7.73 × 10^−9^
cell periphery (GO:0071944)	1888	98	63.26	+	1.55	7.45 × 10^−3^
plasma membrane (GO:0005886)	1834	95	61.45	+	1.55	1.15 × 10^−2^
intrinsic component of membrane (GO:0031224)	3244	166	108.69	+	1.53	2.94 × 10^−6^
integral component of membrane (GO:0016021)	3200	163	107.21	+	1.52	6.18 × 10^−6^
Membrane(GO:0016020)	4745	238	158.98	+	1.50	3.01 × 10^−10^
Cell (GO:0005623)	7948	328	266.29	+	1.23	4.09 × 10^−5^
cell part (GO:0044464)	7895	325	264.52	+	1.23	7.49 × 10^−5^
Intracellular (GO:0005622)	6948	279	232.79	+	1.20	3.08 × 10^−2^
intracellular ribonucleoprotein complex GO:0030529)	437	2	14.64	-	<0.20	4.24 × 10^−2^
ribonucleoprotein complex (GO:1990904)	438	2	14.67	-	<0.20	4.12 × 10^−2^
Unclassified	6062	115	203.10	-	0.57	0.00 × 10
**GO molecular function complete**						
oxidoreductase activity (GO:0016491)	486	38	16.28	+	2.33	3.68 × 10^−3^
ion binding (GO:0043167)	3114	144	104.33	+	1.38	3.63 × 10^−2^
Unclassified	6653	155	222.9	-	0.70	0.00 × 10

DE turkey genes were matched to the chicken gene reference list for analysis in PANTHER [38]. For each, Gene Ontology category, the number of genes in the reference list and those differentially expressed in the turkey are given. Fold enrichment is the number of DE genes divided by Expected. *p*-values are as determined by the binomial statistic.

**Table 5:** Summary of PANTHER Overrepresentation Test of the 136 unique differentially expressed (DE) genes in livers of Eastern wild turkeys after AFB_1_ exposure as compared to domesticated turkey.

GO Biological Process Complete	*Gallus gallus*—REFLIST Genes (15782)	Observed Turkey Genes	Expected	over/under	Fold Enrichment	*p*-Value
L-serine biosynthetic process (GO:0006564)	3	3	0.01	+	>100	6.60 × 10^−4^
blood coagulation, fibrin clot formation (GO:0072378)	5	3	0.02	+	>100	3.04 × 10^−3^
plasminogen activation (GO:0031639)	5	3	0.02	+	>100	3.04 × 10^−3^
positive regulation of heterotypic cell-cell adhesion (GO:0034116)	6	3	0.02	+	>100	5.24 × 10^−3^
L-serine metabolic process (GO:0006563)	6	3	0.02	+	>100	5.24 × 10^−3^
zymogen activation (GO:0031638)	9	4	0.03	+	>100	1.15 × 10^−4^
regulation of heterotypic cell-cell adhesion (GO:0034114)	9	3	0.03	+	>100	1.76 × 10^−2^
fibrinolysis (GO:0042730)	9	3	0.03	+	>100	1.76 × 10^−2^
negative regulation of endothelial cell apoptotic process (GO:2000352)	11	3	0.03	+	87.84	3.20 × 10^−2^
neg regulation of ext apoptotic signaling pathway (GO:1902042)	11	3	0.03	+	87.84	3.20 × 10^−2^
positive regulation of vasoconstriction (GO:0045907)	12	3	0.04	+	80.52	4.14 × 10^−2^
positive regulation of peptide hormone secretion (GO:0090277)	30	4	0.09	+	42.94	1.36 × 10^−2^
cell-matrix adhesion (GO:0007160)	44	5	0.14	+	36.6	1.52 × 10^−3^
coagulation (GO:0050817)	65	5	0.20	+	24.78	1.02 × 10^−2^
blood coagulation (GO:0007596)	65	5	0.20	+	24.78	1.02 × 10^−2^
cell-substrate adhesion (GO:0031589)	66	5	0.20	+	24.4	1.10 × 10^−2^
hemostasis (GO:0007599)	66	5	0.20	+	24.4	1.10 × 10^−2^
positive regulation of protein secretion (GO:0050714)	69	5	0.21	+	23.34	1.36 × 10^−2^
positive regulation of peptide secretion (GO:0002793)	78	5	0.24	+	20.65	2.46 × 10^−2^
alpha-amino acid metabolic process (GO:1901605)	104	6	0.32	+	18.58	4.71 × 10^−3^
regulation of response to external stimulus (GO:0032101)	264	8	0.82	+	9.76	7.87 × 10^−3^
small molecule metabolic process (GO:0044281)	816	13	2.53	+	5.13	4.46 × 10^−3^
single-organism metabolic process (GO:0044710)	1775	19	5.51	+	3.45	3.17 × 10^−3^
single-organism process (GO:0044699)	6214	39	19.29	+	2.02	5.62 × 10^−5^
Unclassified (UNCLASSIFIED)	6196	6	19.24	-	0.31	0.00 × 10

DE turkey genes were matched to the chicken gene reference list for analysis in PANTHER [38]. For each, Gene Ontology category, the number of genes in the reference list and those differentially expressed in the turkey are given. Fold enrichment is the number of DE genes divided by Expected. *p*-values are as determined by the binomial statistic.

**Table 6:** Differential expression (DE) of genes from major enzyme groups responsible for metabolizing xenobiotic chemicals.

ID	EW AFB vs. CNTL		DT AFB vs. CNTL		CNTL EW vs. DT		AFB EW vs. DT		Description
	FDR Pval	log_2_FC	FDR Pval	log_2_FC	FDR Pval	log_2_FC	FDR Pval	log_2_FC	
*AKR1D1*	0.9964	0.0559	0.0000	−2.6518	0.0000	−3.2445	0.2276	−0.5988	aldo-keto reductase family 1, member D1
*ALDH2*	0.0190	−0.8404	0.0000	−1.5779	0.1522	−0.5354	0.9648	0.1539	aldehyde dehydrogenase 2 family (mitochondrial)
*AOX1*	0.0000	2.1137	0.0010	0.8410	0.0574	−0.6975	0.2037	0.5258	aldehyde oxidase 1
*COMT*	0.0000	−2.5729	0.0000	−2.8723	0.0373	−0.7682	0.2064	−0.5319	catechol-*O*-methyltransferase
***CYP1A5***	0.0000	−6.0043	0.0000	−9.2792	0.0721	−0.8105	0.0000	2.4042	cytochrome P450, family 1, subfamily A, polypeptide 5
***CYP3A37***	0.0640	−0.9576	0.0000	−2.8884	0.0007	−1.5099	0.8268	0.3698	cytochrome P450 3A37
*CYP3A80*	0.9731	−0.1270	0.0052	−1.0866	0.0022	−1.7031	0.3001	−0.8022	cytochrome P450 3A80
*EPHX1*	0.0000	3.0994	0.0007	0.9636	0.2271	−0.8719	0.0009	1.2112	epoxide hydrolase 1, microsomal (xenobiotic)
*EPHX2*	0.0000	−2.7512	0.0000	−2.5788	0.4757	0.4301	0.8342	0.2075	epoxide hydrolase 2, cytoplasmic
*EPHX4*	0.0000	3.1365	0.0091	2.4479	1.0000	0.3859	0.1409	1.0154	epoxide hydrolase 4
*GSTA1.1* *	0.0000	−2.0007	0.0000	−2.6584	0.0066	1.3021	0.0000	1.9010	glutathione *S*-transferase alpha class A1.1
*GSTA1.3* *	0.0004	−1.0804	0.0000	−1.6746	0.1740	0.6098	0.0003	1.1419	glutathione *S*-transferase alpha class A1.3
*GSTA2*	0.0000	−1.7332	0.0000	−1.9211	0.1019	0.5386	0.0098	0.6713	glutathione *S*-transferase 2
***GSTA3***	0.8469	0.1297	0.0014	1.4667	0.0000	2.3130	0.0063	0.9211	glutathione *S*-transferase 3
***GSTA4***	0.0000	−5.9578	0.0000	−6.4058	0.3086	0.5690	0.0204	0.9569	glutathione *S*-transferase 4
*GSTK1*	0.0000	−1.8072	0.0000	−2.9850	0.1814	−0.5583	0.0773	0.5713	glutathione *S*-transferase kappa 1
*GSTZ1*	0.0000	−3.3639	0.0000	−4.1131	1.0000	0.0517	0.0325	0.7477	glutathione *S*-transferase zeta 1
*LOC100538434*	0.0000	1.7178	0.7570	0.1451	0.0038	−0.9746	0.3530	0.5480	cytochrome P450 4B1-like
*LOC100538440*	0.0000	−2.8970	0.0000	−2.2682	0.1163	0.5550	0.9682	−0.1256	glutathione *S*-transferase theta-1-like
*LOC100538588*	0.0025	−1.1881	0.0000	−3.4884	0.0001	−1.2845	0.0758	0.9622	cytochrome P450 4B1-like
*LOC100538595*	0.0000	−7.0046	0.0000	−8.5660	1.0000	0.0523	0.1895	1.5495	glutathione *S*-transferase theta-1
*LOC100542486*	0.0000	−3.3050	0.0000	−7.4005	0.1187	−0.8954	0.0000	3.1496	cytochrome P450 1A4
*LOC100543147*	0.0000	−1.9129	0.0001	−1.0244	0.0097	0.8235	0.9927	−0.1181	cytochrome P450 2U1
*LOC100543474*	0.0000	4.4089	0.0000	3.2044	0.6256	−0.4228	0.0227	0.7294	glutathione *S*-transferase omega-1-like
*LOC100544448*	0.0000	−3.1080	0.0000	−3.1865	1.0000	−0.1757	1.0000	−0.1579	cytochrome P450 2C9-like
*LOC100544938*	0.0000	−1.6254	0.0052	−0.8255	0.8684	0.3729	0.3538	−0.4894	cytochrome P450 26B1
*LOC100545163*	0.0000	−2.7394	0.0000	−3.5785	0.0404	−0.7668	1.0000	0.0207	alcohol dehydrogenase 1
*LOC100545251*	0.0055	−0.8765	0.0000	−1.7618	0.6466	0.3201	0.0085	1.1514	sulfotransferase 1C1-like
*LOC100545313*	0.0011	−6.6206	0.0000	−12.9085	0.0662	2.5030	0.0071	9.2625	sulfotransferase 6B1-like
*LOC100545337*	0.0042	−0.8258	0.4111	0.3711	0.0116	1.3434	1.0000	0.0879	sulfotransferase family cytosolic 2B member 1-like
*LOC100545469*	0.0000	−7.4792	0.0000	−12.4538	0.8899	−0.3159	0.0330	4.6071	sulfotransferase 6B1-like
*LOC100545683*	0.0000	−2.4276	0.0000	−5.5488	0.3422	−0.8455	0.0915	2.2195	cytochrome P450 2H1-like
*LOC100546724*	0.0000	1.8339	0.0095	0.8064	0.1178	−0.9930	1.0000	−0.0121	cytochrome P450 2K4-like
*LOC100546874*	0.0169	0.7907	0.0000	0.9012	1.0000	0.0100	0.9251	−0.1538	cytochrome P450 2W1-like
*LOC100547030*	0.0000	−7.6458	0.0000	−13.6234	1.0000	−0.2262	0.0010	5.8718	cytochrome P450 2W1-like
*LOC100547576*	0.0000	−4.3115	0.0000	−5.2719	0.0014	−1.0131	1.0000	−0.1127	UDP-glucuronosyltransferase 1-1-like
*LOC100547627*	0.7689	0.1433	0.0094	−0.7229	0.0023	−1.0592	0.7311	−0.2480	sulfotransferase family cytosolic 1B member 1
*LOC100547794*	0.0284	0.7020	0.9613	0.0689	0.9308	−0.3488	0.7143	0.2327	cytochrome P450 2J2-like
*LOC100547885*	0.0011	−0.9841	0.0000	−2.4195	0.0453	−0.6672	0.0321	0.7153	UDP-glucuronosyltransferase 1-1-like
*LOC100548173*	0.0000	3.7249	0.0000	2.5969	1.0000	−0.3391	0.1461	0.7080	galactosylgalactosylxylosylprotein 3-betaglucuronosyltransferase 1-like
*LOC100548279*	0.0000	−7.9193	0.0000	−11.0735	0.5445	−0.5533	0.0906	2.5242	cytochrome P450 2K4-like
*LOC100548322*	0.0000	−5.6388	0.0000	−7.6409	0.0002	−1.2226	0.0429	0.7206	cytochrome P450 2D17
*LOC100548433*	0.0000	−5.3627	0.0000	−8.5915	1.0000	−0.1464	0.0000	3.0249	cytochrome P450 2K1-like
*LOC100548965*	0.0027	1.2412	0.4561	0.2791	0.5805	0.4725	0.0144	1.3798	cytochrome P450 2J3-like
*LOC100549160*	0.0000	−1.5607	0.0000	−1.7517	0.0335	−0.7103	0.3717	−0.5706	cytochrome P450 4F22
*LOC100549268*	0.0000	2.8734	0.0067	1.6835	1.0000	−0.3799	0.2658	0.7421	aldehyde oxidase 2-like
*LOC100549312*	0.3177	0.3574	0.0000	−1.5738	0.0000	−1.9158	1.0000	−0.0363	UDP-glucuronosyltransferase 2C1-like
*LOC100549991*	0.0000	−1.7655	0.0000	−1.6943	0.0002	1.3894	0.0039	1.2611	arylamine *N*-acetyltransferase, liver isozyme
*LOC100550430*	0.0512	−0.6659	0.0000	−1.3736	0.2945	−0.4855	0.9495	0.1737	cytochrome P450 4F22
*LOC104909216*	0.0925	3.6677	0.0207	4.6262	1.0000	0.0000	0.5908	−1.0481	cytochrome P450 2J6-like
*LOC104909734*	0.0000	−6.4443	0.0023	−2.1011	0.0000	4.1441	1.0000	−0.2356	sulfotransferase family cytosolic 2B member 1-like
*LOC104910746*	0.0000	−3.6385	0.0000	−5.1988	0.7764	−0.3620	0.1896	1.1405	alcohol dehydrogenase 1-like
*LOC104911955*	0.3696	−0.4051	0.0000	−1.7954	0.0045	−1.2985	1.0000	0.0458	cytochrome P450 2H2
*LOC104912373*	0.0000	6.1224	0.0000	7.7909	0.2163	2.2765	0.2724	0.5912	sulfotransferase 6B1-like
*LOC104912427*	0.0001	0.8626	0.0006	0.8049	0.7651	−0.3468	0.3464	−0.3463	cytochrome P450 2J2-like
*LOC104912428*	0.1738	0.5765	1.0000	0.0281	0.2879	0.5209	0.0401	1.0161	cytochrome P450 2J2-like
*LOC104912494*	0.0006	1.5962	0.0373	0.5871	1.0000	−0.1563	0.3537	0.7988	cytochrome P450 2J2-like
*LOC104915391*	0.0000	−4.4041	0.0000	−4.5429	1.0000	0.2453	0.9573	0.3281	sulfotransferase family cytosolic 2B member 1-like
*LOC104915445*	0.0000	−3.6108	0.0000	−4.2325	0.0262	−0.8559	0.9682	−0.2891	alcohol dehydrogenase 1-like
*LOC104915446*	0.0000	−3.8678	0.0000	−5.4661	0.1525	−0.6518	0.4129	0.8857	alcohol dehydrogenase 1-like
*LOC104915473*	0.0000	1.5141	0.8643	0.0874	0.8777	−0.3084	0.0010	1.0674	UDP-glucuronosyltransferase 1-9-like
*LOC104915474*	0.8756	−0.1560	0.0000	−3.7112	0.0000	−2.5312	0.0418	0.9617	UDP-glucuronosyltransferase 1-6-like
*LOC104915476*	0.0006	1.6704	0.9112	0.1504	0.4764	−1.0151	0.6292	0.4445	UDP-glucuronosyltransferase 1-1-like
*LOC104915477*	0.0000	−4.3026	0.0000	−6.1630	1.0000	0.0675	0.0160	1.8961	UDP-glucuronosyltransferase 1-6 pseudogene
*LOC104915478*	0.0000	−1.7304	0.0000	−3.7892	1.0000	0.1939	0.0017	2.2036	UDP-glucuronosyltransferase 1-1-like
*LOC104915479*	0.0000	−2.4027	0.0000	−5.5537	0.1838	−0.9621	0.0384	2.1351	cytochrome P450 2H1-like
*LOC104915586*	0.0000	−5.9323	0.0090	−2.7687	0.0023	3.1518	1.0000	−0.0267	sulfotransferase family cytosolic 2B member 1-like
*LOC104915609*	0.0022	−3.8661	0.0686	−3.9012	0.8037	1.2048	1.0000	1.5781	cytochrome P450 2C19-like
*LOC104915610*	0.0000	3.8631	0.0000	3.5278	0.9437	−0.6231	0.9255	−0.3368	cytochrome P450 2C4-like
*LOC104916399*	0.0000	−6.0701	0.0000	−7.5516	0.5648	0.6141	0.4295	2.0569	cytochrome P450 2C27-like
*LOC104916553*	0.0000	−4.9703	0.0000	−3.9795	0.5369	0.6853	0.9367	−0.3716	sulfotransferase family cytosolic 2B member 1-like
*LOC104916909*	0.0000	−5.6085	0.0000	−7.0458	0.5315	0.5373	0.1227	1.9411	cytochrome P450 2C31-like
*NQO1*	0.7812	−0.1408	0.0008	−0.8283	1.0000	−0.1435	0.2145	0.4945	NAD(P)H dehydrogenase, quinone 1
*NQO2*	0.0000	−0.9071	0.4267	−0.2242	0.4389	0.3990	0.5130	−0.3430	NAD(P)H dehydrogenase, quinone 2
*PTGS1*	0.0000	1.4444	0.1729	0.5413	0.9517	−0.4782	0.5266	0.3552	prostaglandin-endoperoxide synthase 1
*PTGS2*	0.7963	0.1315	0.0000	1.3365	0.1157	0.7544	0.1495	−0.5069	prostaglandin-endoperoxide synthase 2
*SULT4A1*	0.1141	−0.6315	0.0039	−0.8113	1.0000	0.3027	0.4515	0.4192	sulfotransferase family 4A, member 1
*SULT6B1*	0.0457	−1.6954	0.0001	−2.6041	1.0000	0.0540	0.7545	0.9577	sulfotransferase family, cytosolic, 6B, member 1
*TPMT*	0.9701	0.0725	0.0017	−0.7106	0.8466	−0.2968	0.2312	0.4402	thiopurine S-methyltransferase
*UGT8*	0.0000	1.7926	0.0000	1.1497	0.5752	−0.3194	0.6356	0.2705	UDP glycosyltransferase 8

Genes included were significant (FDR *p*-value < 0.05) in at least one comparison. Comparisons highlighted in green are down regulated and those in red up regulated. Cytochrome P450 (CYP) and glutathione S-transferase (GST) family members shown to have *in vitro* activity towards AFB_1_ and its metabolites in turkey are indicated in bold. * Due to similarity, these likely include transcripts assignable to *GSTA1.1*, *A1.2*, and *A1.3*.

## Supplementary Materials

The following are available online at www.mdpi.com/2072-6651/10/1/42/s1. Figure S1: Hierarchical clustering of samples based on Euclidean distance reiterated relationships shown by PCA. Figure S2: Distribution of genes expressed in turkey liver by treatment group. Figure S3: Differential fold change in DEGs shared by Eastern wild (EW) and domesticated (DT) birds exposed to AFB_1_ in comparison to controls. Table S1. Mean quality-trimmed RNA-seq read counts for genes with mapped reads in the livers of wild and domesticated turkeys. Table S2. Summary of pairwise differential gene expression analysis of liver transcriptomes. Table S3. Fifty genes showing the greatest differential expression in each pairwise comparison of treatment groups. Table S4. Summary of PANTHER Overrepresentation test of the 3380 differentially expressed genes shared in the AFB_1_-treated birds as compared to controls. Table S5. Significant differentially expressed genes (FDR *p*-values < 0.05 and |log_2_FC| > 2.0) identified in each pairwise comparison of genetic groups (Eastern wild and domesticated turkey).

## Abbreviations

AFB_1_	aflatoxin B_1_
AFBO	exo-AFB1-8,9-epoxide
BB	Broad Breasted White
BW	body weight
Ct	threshold cycle
CYP	cytochrome P450
DE	differentially expressed
DEG	differentially expressed gene
DT	domesticated turkey
EW	Eastern wild turkey (*Meleagris gallopavo silvestris*)
FC	fold change
FDR	false discovery rate
GO	gene ontology
GST	glutathione S-transferase
IPA	Ingenuity Pathway Analysis
lncRNA	long non-coding RNA
miRNA	micro-RNA
ncRNA	non-coding RNA
PCA	principal component analysis
qRT-PCR	quantitative real-time polymerase chain reaction
RGW	Rio Grande wild turkey (*Meleagris gallopavo intermedia*)
