# Supplementary material for: Comparative Response of the Hepatic Transcriptomes of Domesticated and Wild Turkey to Aflatoxin B1
Source: Toxins (Basel). 2018 Jan 13;10(1):42. doi: 10.3390/toxins10010042 (PMC5793129; doi:10.3390/toxins10010042)
Supplement: Supplementary file 1 [file toxins-10-00042-s001.zip › toxins-252543-supplementary proof/toxins-252543-supplementary proof.pdf]

Supplementary Materials: Comparative Response of the Hepatic Transcriptomes of Domesticated and Wild Turkey to Aflatoxin B_1

Kent M. Reed, Kristelle M. Mendoza, Juan E. Abrahante and Roger A. Coulombe

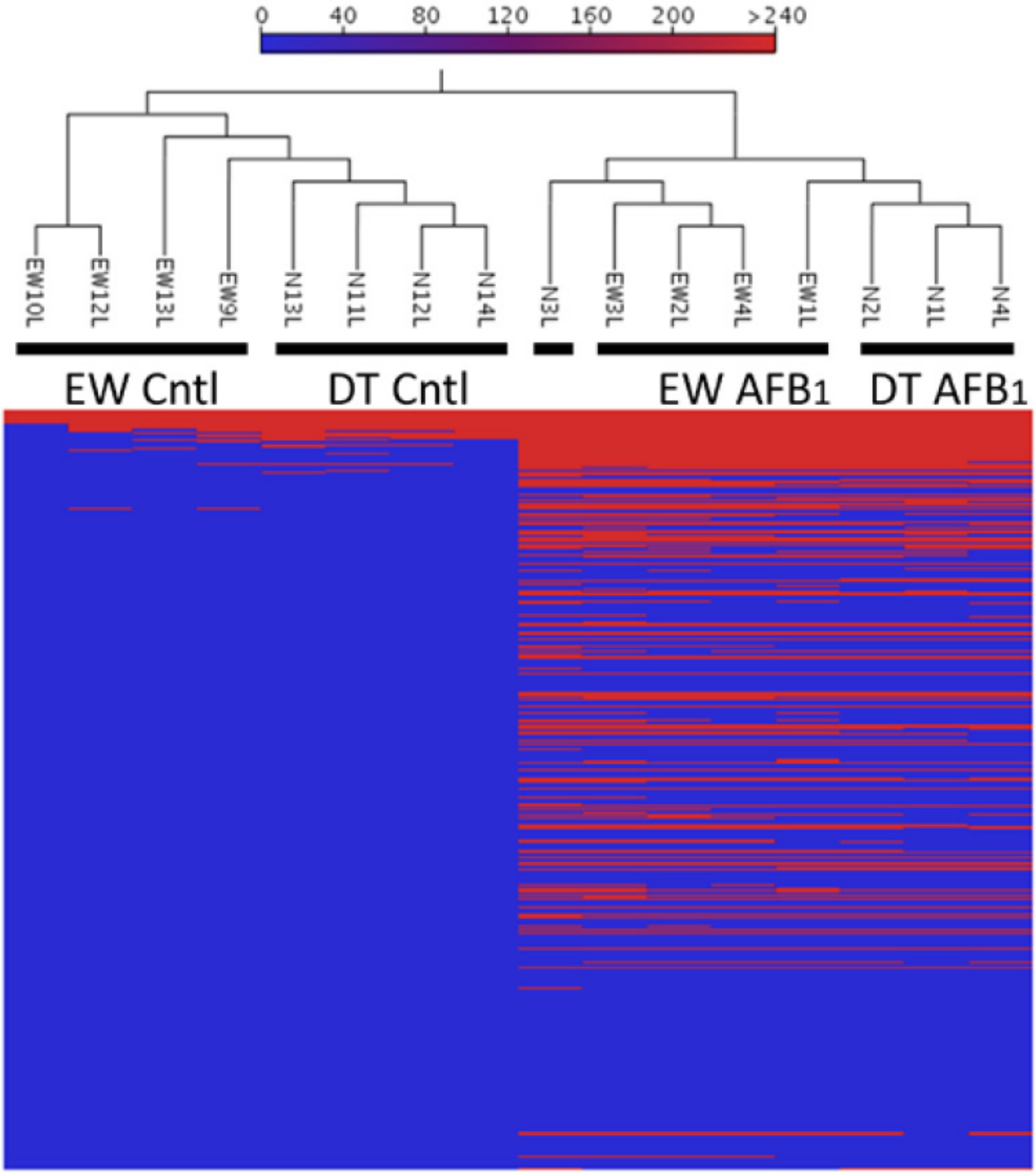

Figure S1. Hierarchical clustering of samples based on Euclidean distance reiterated relationships shown by PCA. Global gene expression differences among groups are illustrated in the heat map constructed from the 500 co-expressed genes with the greatest experiment-wise differences in averages normalized expression.

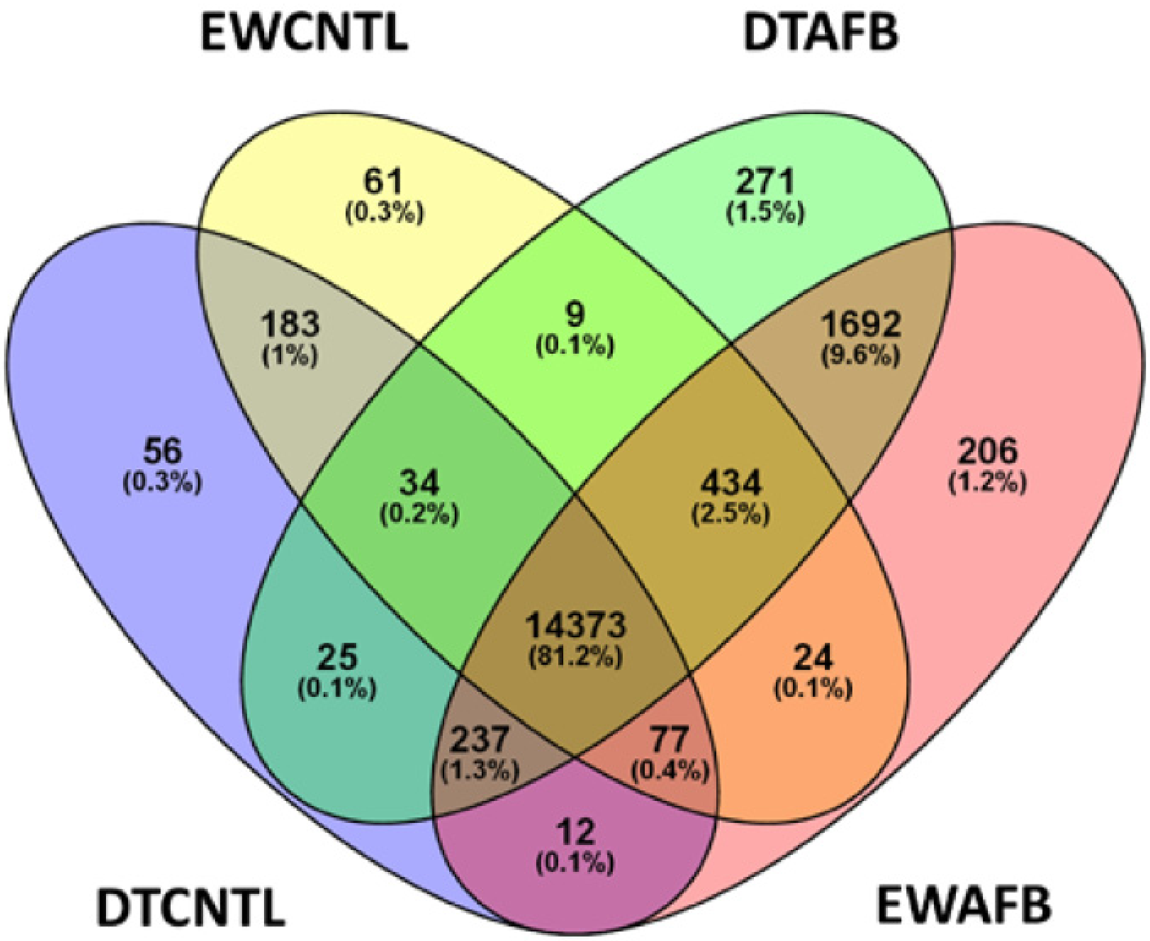

Figure S2. Distribution of genes expressed in turkey liver by treatment group. Number and precent of genes for which the average number of mapped reads per group was ≥ 3.0 are included. EWCNTL = Eastern wild control, DTCNTL = domesticated control, EWAFB = Eastern wild aflatoxin B1 and DTAFB = domesticated aflatoxin B1.

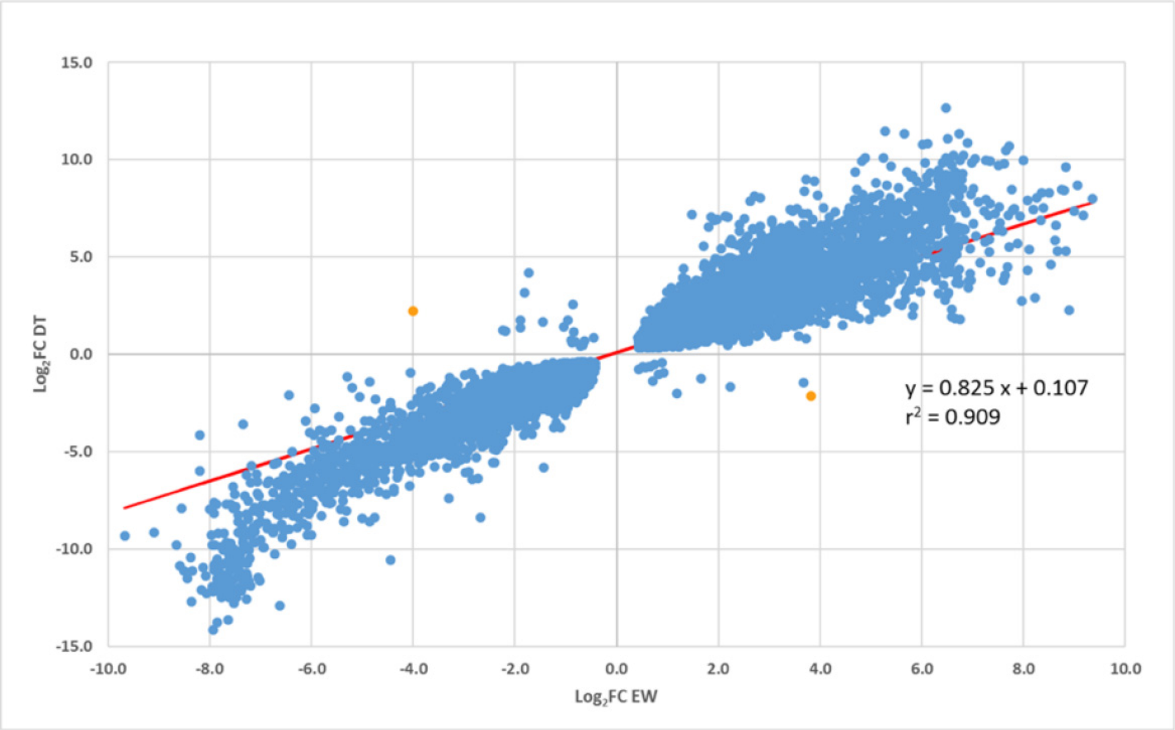

Figure S3. Differential fold change in DEGs shared by wild (EW) and domesticated (DT) birds exposed to AFB_1 in comparison to controls. The best-fitting regression line ($r^2 = 0.909$) is indicated in red. Yellow points indicate two genes showing statistically significant differential expression (FDR *p*-value < 0.05) with opposite directional change ($log_2FC > 2.0$).
